# Sodium Nitroprusside Changed The Metabolism of Mesenchymal Stem Cells to An Anaerobic State while Viability and Proliferation Remained Intact

**DOI:** 10.22074/cellj.2016.4875

**Published:** 2016-12-21

**Authors:** Sadiyeh Pari, Mohammad Hussein Abnosi, Reza Pakyari

**Affiliations:** Department of Biology, Faculty of Sciences, Arak University, Arak, Iran

**Keywords:** Cell Survival, Lactate Dehydrogenase, Mesenchymal Stem Cells, Morphology, Nitroprusside

## Abstract

**Objective:**

We used sodium nitroprusside (SNP), a nitric oxide (NO) releasing molecule, to understand its effect on viability and proliferation of rat bone marrow mesenchymal stem cells (BM-MSCs).

**Materials and Methods:**

This experimental study evaluated the viability and morphology of MSCs in the presence of SNP (100 to 2000 µM) at 1, 5, and 15 hours. We chose the 100, 1000, and 2000 µM concentrations of SNP for one hour exposure for further analyses. Cell proliferation was investigated by the colony forming assay and population doubling number (PDN). Na^+^, K^+^, and Ca^2+^ levels as well as activities of lactate dehydrogenase (LDH), alkaline phosphatase (ALP), aspartate transaminase (AST), and alanine transaminase (ALT) were measured.

**Results:**

The viability of MSCs dose-dependently reduced from 750 µM at one hour and 250 µM at 5 and 15 hours. The 100 µM caused no change in viability, however we observed a reduction in the cytoplasmic area at 5 and 15 hours. This change was not observed at one hour. The one hour treatment with 100 µM of SNP reduced the mean colony numbers but not the diameter when the cells were incubated for 7 and 14 days. In addition, one hour treatment with 100 µM of SNP significantly reduced ALT, AST, and ALP activities whereas the activity of LDH increased when incubated for 24 hours. The same treatment caused an increase in Ca^2+^ and reduction in Na^+^ content. The 1000 and 2000 µM concentrations reduced all the factors except Ca^2+^ and LDH which increased.

**Conclusion:**

The high dose of SNP, even for a short time, was toxic. The low dose was safe with respect to viability and proliferation, especially over a short time. However elevated LDH activity might increase anaerobic metabolism.

## Introduction

Nitric oxide (NO) is a hydrophobic, small diatomic molecule with a high diffusion coefficient (4.8×10^5^cm^2^/second in H_2_O) (1). NO is an industrial byproduct that can be produced naturally during the electrical discharges of lightning in thunderstorms (2). NO is highly reactive in nature and can react with metal complexes or other radicals. This molecule also affects the biological molecules such as DNA, proteins, and lipids as a reactive NO species (3). The NO molecule is quite stable under physiological conditions and can move freely across cell membranes, which within seconds may reach distances of 200 μm in tissues (4).

In cells, NO is produced by three isoforms of nitric oxide synthase (NOS) endothelial NOS (eNOS), inducible NOS (iNOS), and neuronal NOS (nNOS) (5). All utilize L-arginine and molecular oxygen as substrates (6). NO plays a variety of roles in biological systems such as regulation of cell growth, survival, apoptosis, proliferation, and differentiation at the cellular level (7).

For therapeutic purposes, NO donors such as nitroglycerine, isosorbide mononitrate, and sodium nitroprusside (SNP) (8) are used to induce NO-related activities (9). SNP has been used in clinical practice for 40 years, particularly for cardiovascular complications as an arterial and venous vasodilator (10). After libration of NO, this molecule via cyclic cyclic guanosine monophosphate (cGMP) (11) causes the reduction of intracellular Ca^2+^and increases K^+^ channel permeability (12) which finally acts as a vasodilator to reduce blood pressure. In addition to the NO producing activity, SNP when interact with oxyhemoglobin in the blood releases cyanide (CN) which inhibits aerobic metabolism by inhibiting the final step of oxidative phosphorylation (13).

Chen et al. (14) reported that treatment of osteoblasts with 1.5 mM SNP caused 29% cell death. The 2 mM SNP treatment led to 58% cell death, and SNP at <1 mM was not cytotoxic to the cells. In another study, Chen et al. (15) reported that administration of SNP in an osteoblast culture led to DNA fragmentation and reduction of the mitochondrial membrane potential and activity of NADH dehydrogenase (complex I) in a time-dependent manner. In parallel with the mitochondrial dysfunction, SNP also increased levels of intracellular reactive oxygen species (ROS).

Numerous other investigators have derived the same results with respect to the toxic effect of SNP on osteoblasts. They reported significant reductions in cell viability (16), complete blockage of Runx2 expression (4), increased cell apoptotic morphological and nuclear chromatin condensation as well as DNA fragmentation (17) and interaction increased levels of intracellular ROS (18).

## Materials and Methods

### Bone marrow cell culture

In this experimental study, we purchased Wistar rats (6-8 weeks old) from Pasteur Institute (Iran). The animals were maintained in the Animal House of Arak University under standard conditions of light, temperature, and food. The animals were sacrificed according to an animal laboratory protocol approved by Arak University that used excessive chloroform inhalation. Then, under sterile conditions, their femora and tibia were surgically removed and the bone marrow content was extracted in 3 ml of Dulbecco modified Eagle’s medium (DMEM, Gibco, Germany) supplemented with 15% fetal bovine serum (FBS, Gibco, Germany) and penicillin/streptomycin (Gibco, Germany). Bone marrow content was centrifuged at 2500 rpm for 5 minutes, re-suspended in 5 ml culture media, then plated in culture flasks and incubated at 37˚C in an atmosphere of 5% CO_2_. We replaced the medium one day after culture initiation and subsequently the medium was changed twice a week until the bottom of the flask was covered with cells (confluency). The cells were trypsinized (trypsin-EDTA, Gibco, Germany) and passed to another culture flask as the first passage. The cultures were subsequently expanded through two additional subcultures at which the cells were used for further investigation.

### Exposure to sodium nitroprusside 

The cells were plated in an appropriate culture dish and allowed to attach for 24 hours. Then, in the presence of the control group, the treated cells were exposed to 100, 250, 500, 750, 1000, 1250, 1500, 1750 or 2000 µM of SNP (Merck, Germany). Measurements for each analysis were repeated three times in a bracket model.

### Cell viability assays

#### Trypan blue exclusion assay 

MSCs were seeded at a density of 50000 cells per well in 24-well culture plates and contaminated culture media that contained different concentrations of SNP were added to the respective wells. After treating the cells for 1, 5, and 15 hours, we added fresh culture media and the plates were incubated for an additional 24 hours. We used PBS to wash the cells after which they were harvested with trypsin/EDTA and subsequently collected by centrifugation at 2500 rpm for 5 minutes. Following centrifugation, the cells were re-suspended in fresh culture media, then 50 μl of the cell suspension was mixed and incubated for 2 minutes at 37˚C with an equal volume of trypan blue (Sigma, America). We used a hemocytometer chamber to count the total number of viable cells. The experiment was carried out in triplicate and the results expressed as a percentage of the viable cells.

#### 3-(4,5-dimethy thiazole-2-yl)-2,5 diphenyltetrazolium (MTT) assay 

We used the 3-(4,5-dimethy thiazole-2-yl)-2,5 diphenyltetrazolium (MTT) assay to determine cell viability. MSCs, at a density of 15000 cells per well, were cultured in a microplate after which treatment with SNP was carried out in the same manner as the previous test. Next, the cells were washed with PBS and 10 μl of MTT/100 μl of FBS-free culture media was added. The plates were incubated for 4 hours. Once the yellow soluble tetrazolium converted to blue formazan, the resultant crystals were dissolved in 100 μl of dimethyl sulfoxide (DMSO, Sigma, USA) and absorbance was measured at 505 nm with an ELISA reader (SCO Diagnostic, Germany). A standard graph was plotted. We calculated the viable cells using the linear formula: Y=0.013X+0.007 with R^2^=0.996, where Y was the absorbance and X was the number of viable cells.

Based on the viability results, we chose the 100, 1000, and 2000 µM of SNP as representative of a broad range of concentrations. We selected the one hour treatment time as the lower toxicity time for further analysis. Although the one hour treatment time was the same for each analysis, the incubation periods differed. 

#### Quantification of proliferation ability

After the third passage, we performed the colony forming assay and calculated the population doubling number (PDN) in order to quantify the cell proliferation ability.

### Colony forming assay 

In this assay, 5×10^4^cells were separately seeded in 3 cm sterile plates. The cells were treated with 100, 1000, and 2000 µM of SNP for one hour. The plates were subsequently washed and supplied with fresh media. These plates were incubated for 7 and 14 days, with media replacement every three days. After washing with PBS, crystal violet stain (0.5 g crystal violet in 100 ml methanol solution) was added and the plates were incubated at room temperature for 15 minutes. We used a light microscope equipped with a graticule eyepiece to estimate the diameter (μm) and colony numbers.

### Population doubling number 

In order to estimate the PDN, 5×10^4^cells were separately seeded in 3 cm sterile plates. After treating the cells with 100, 1000, and 2000 µM of SNP for one hour, the plates were washed and supplied with fresh media, then incubated for 5, 10, and 15 days. We replaced the culture media every three days. Plates were washed with PBS and then the cells were harvested with trypsin-EDTA. We used a hemocytometer chamber to count the number of cells. We determined the PDN of these cells according to the following equation: $\mathrm{PDN}=\frac{\mathrm{logN}}{\mathrm{N0}}\times 3.31$ where N0 was the initial number of cells seeded and N was the number of cells harvested after 5, 10, and 15 days.

### Morphology

MSCs that attached to 12-well plates were treated with 100, 1000, and 2000 µM of SNP for 1, 5, and 15 hours followed by the addition of fresh culture media and an additional incubation period of 24 hours. The plates were washed with PBS and 10 μl of Hoechst (50 µg/ml, Sigma, USA) was added, after which the plates were subsequently incubated for 15 minutes at room temperature. In addition, we analyzed the cell cytoplasm following incubation for 2 minutes with 10 μl of acridine orange (5 µg/ml) in a separate chamber. The cells were observed under an inverted fluorescence microscope (Olympus, IX70) equipped with a camera (DP72) at ×200 magnification. The nuclei diameter and cytoplasm area of the cells were measured in μm using Motic Image software (Micro Optical Group Company version 1.2).

### Extracting the cell content

Control cells and experimental cells treated with 100, 1000 and 2000 µM of SNP for one hour were washed with PBS and incubated for 24 hours. Then, the cells were harvested with trypsin/EDTA follow by centrifugation at 2500 rpm for 5 minutes. The cells were washed twice with tris-HCl NaCl and subsequently maintained at -20˚C overnight in lysis buffer (20 mM tris-HCl, pH=7.2) in order to break the cell membrane. Finally the homogenate was thawed and centrifuged at 12000 g for 10 minutes in order to extract the cell content. We used the Lowry method to estimate the total protein content of each sample. A standard graph was plotted using bovine serum albumin (BSA) and the linear formula Y=0.001X+0.063, with R^2^=0.990 in order to calculate the concentration of the unknown protein samples. In the formula, Y represented the absorbance and X the concentration (μg) of the protein in each sample.

### Enzyme activity determination

We determined the activities of the enzymes alanine transaminase (ALT), aspartate transaminase (AST), and lactate dehydrogenase (LDH) in protein lysate using a commercial kit (Pars Azmoon, Iran) according to manufacturer’s instructions. Absorbance was measured at 340 nm using a spectrophotometer (T80+, PG Instrument Ltd., England) based on an equal amount of protein.

### Determination of alkaline phosphatase activity 

Alkaline phosphatase (ALP) activity was determined using a spectrophotometer (T80+, PG Instrument, Ltd., England). The enzymatic activity was carried out in protein lysate based on an equal amount of protein using p-nitrophenyl phosphate (pNPP) as the substrate according to the kit’s instructions (Pars azmoon, Iran). The instrument absorbance was adjusted to 410 nm and the sample measurement was carried out in the presence of a blank.

### Intracellular Ca^2+^assay

We used a commercial kit (Pars Azmoon, Iran) to determine the amount of Ca^2+^ The developed color was measured at 570 nm using a spectrophotometer (T80+, PG Instrument, Ltd., England). A standard graph was plotted using a different solution of CaCl_2_ and the amount of Ca^2+^in the cell extract was estimated using the linear formula Y=0.0763X-0.0039 with R^2^=0.998 where Y represented absorbance and X was the concentration of Ca^2+^in the cell extract.

### Determination of Na^+^and K^+^levels

We have estimated the amounts of Na^+^and K^+^in the cell extract using a flame photometer (Model PFP7, England). In a flame photometer, Na^+^and K^+^ emit light of different wavelengths. The emission can be measured using appropriate filters, which is correspondent to the respective concentrations. We used the same instrument and different concentrations of NaCl and KCl to plot a standard graph. The linear formula Y=0.005X+0.0592 with R^2^=0.992 where Y=0.0201X+0.0039 with R^2^=0.996 were obtained for Na^+^and K^+^. Here, Y represented the absorbance, where X was the concentration of each one of the electrolytes. 

### Statistical analysis 

Data were analyzed with SPSS software, using one-way ANOVA and the Tukey test. The level of significance was P<0.05.

## Results

### Cell viability

Based on trypan blue assay there was a time dependent significant reduction in cell viability. 

Treatment of the cells with SNP caused a significant reduction (P<0.05) from 750 µM at one hour, to 250 µM at 5 hours, and 100 µM at 15 hours compared to the control. However, SNP treatment did not show any significant difference (P>0.05) with 100 to 500 µM at one hour when compared to the control and each other.

After 5 hours of treatment, no significant differences (P>0.05) existed between the 1000 to 1500 µM and 1750 to 2000 µM concentrations with respect to each other. After 15 hours treatment of treatment, we observed no significant difference (P>0.05) between the 1000 to 2000 µM concentrations. However, a highly significant difference (P<0.001) existed with respect to the control (Table 1).

Viability of MSCs according to the MTT assay also confirmed the results from trypan blue staining. Cells treated for one hour showed no significant differences between the 100 to 500 µM concentrations compared to the control group. We observed significant differences (P<0.05) from the 750 to 2000 µM concentrations compared to the control. We observed a significant difference from 100 to 2000 µM at 5 and 15 hours of treatment (Table 2). 

### Colony forming assay 

**Fig.1:**
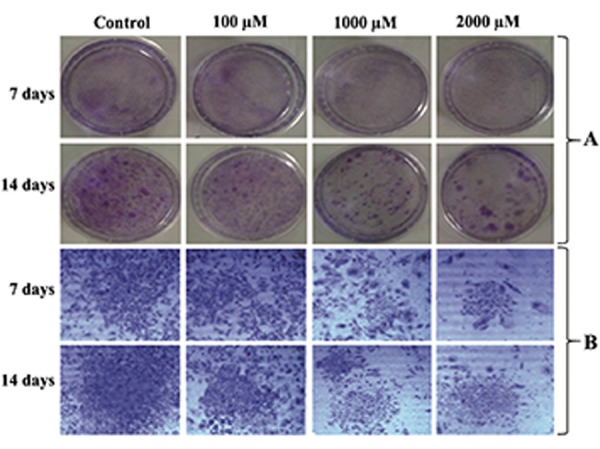
Colony forming assay. A. Culture plates showing the visual difference between the numbers and diameter (mm) of the colonies in the control and treated groups and B. Microscopic photograph showing the visual differences between the colony size in control and treated groups (magnification: ×10).

Treatment of the cells with 1000 and 2000 µM caused highly significant reduction (P<0.001) in numbers and diameters of the colonies at days 7 and 14. Although the numbers of colonies significantly reduced with 100 µM of SNP, there were no significant changes observed in colony diameters at 7and 14 days. The changes in numbers and diameters of the colonies were both significant and dose dependent (Table 3). Macroscopic and microscopic observation of colonies also confirmed the reduction in numbers and diameters of the colonies in the groups treated with 1000 and 2000 µM of SNP (Fig.1A, B).

### Population doubling number

SNP at the 1000 and 2000 µM concentrations significantly reduced (P<0.05) MSC PDN at days 5, 10, and 15 compared to the control. There were no significant differences in PDN at the 100 µM concentration on days 5, 10, and 15 (P>0.05). The data indicated a dose dependent reduction in PDN (Table 4).

**Table 1:** Viability of mesenchymal stem cells (MSCs) after one, 5, and 15 hours of treatment with various concentrations of sodium nitroprusside (SNP) followed by 24 hour incubation based on trypan blue staining


Hour Dose (µM)	1	5	15

0	95.33^a^ ± 0.57	96.67^a^ ± 0.57	95.33^a^ ± 0.57
100	94.00^a^ ± 0.00	92.00^ab^ ± 2.00	90.33^b^ ± 1.15
250	94.00^a^ ± 2.00	88.00^b^ ± 0.00	82.00^c^ ± 1.00
500	92.00^a^ ± 1.37	31.00^c^ ± 0.00	22.00^d^ ± 2.00
750	83.00^b^ ± 1.00	11.33^d^ ± 0.57	03.67^e^ ± 1.15
1000	71.00^c^ ± 1.00	9.00^de^ ± 1.00	00.33^f^ ± 0.57
1250	67.00^cd^ ± 1.00	6.67^e^ ± 1.52	0.00^f^ ± 0.00
1500	58.00^d^ ± 1.00	6.67^e^ ± 2.35	0.00^f^ ± 0.00
1750	27.33^e^ ± 0.57	1.33^f^ ± 0.57	0.00^f^ ± 0.00
2000	13.33^f^ ± 0.50	1.00^f^ ± 0.00	0.00^f^ ± 0.00


Values are means ± SD. Means with the same letter code do not differ significantly from each other within a column (ANOVA, Tukey test, P>0.05).

**Table 2:** Numbers of viable mesenchymal stem cells (MSCs, ×10^3^) after one, 5, and 15 hours of treatment with various concentrations of sodium nitroprusside (SNP) followed by 24 hours of incubation according to the MTT assay


Hour Dose (µM)	1	5	15

0	18.85^a^± 1.61	18.14^a^ ± 1.90	19.43^a^ ± 0.62
100	16.64^ab^ ± 0.64	12.64^b^ ± 1.25	8.24^b^ ± 1.43
250	16.56^ab^ ± 0.81	8.3^c^ ± 0.9	3.90^c^ ± 0.31
500	13.20^bc^ ± 3.7	4.01^d^ ± 0.01	2.90^c^ ± 1.08
750	11.52^c^ ± 1.5	4.08^d^ ± 0.01	2.50^c^ ± 0.4
1000	5.87^d^ ± 0.75	3.12^e^ ± 0.01	2.08^c^ ± 2.03
1250	5.2^d^ ± 1.20	3.04^e^ ± 0.01	1.91^c^ ± 1.87
1500	4.5^d^ ± 0.34	2.20^f^ ± 0.01	1.45^c^ ± 1.51
1750	3.24^d^ ± 1.4	2.47^f^ ± 0.01	1.60^c^ ± 1.47
2000	2.8^d^ ± 1.15	2.06^f^ ± 0.01	1.50^c^ ± 1.46


Values are means ± SD. Means with the same letter code do not differ significantly from each other in a column (ANOVA, Tukey test, P>0.05).

**Table 3:** Proliferation assay shows mean numbers and colony diameters (mm) after one hour of treatment with 100, 1000, and 2000 µM of sodium nitroprusside (SNP) followed by 7 and 14 days of incubation


Day	7		14	
Dose (µM)	Colony numbers	Diameter (mm)	Colony numbers	Diameter (mm)

0	71.5^a^ ± 2.5	2.35^a^ ± 0.53	121.0^a^ ± 2.00		2.73^a^ ± 0.88
100	56.00^b^ ± 3	2.36^a^ ± 01.0	97.00^b^ ± 2.6		2.58^a^ ± 0.55
1000	17.33^c^ ± 2.5	1.98^b^ ± 7.0	34.0^c^ ± 3.5		2.00^b^ ± 06.0
2000	4.30^d^ ± 0.57	1.62^c^ ± 0.8	18.3^d^ ± 5.0		2.30^c^ ± 8.19


Values are means ± SD. Means with the same letter code do not differ significantly from each other in a column (ANOVA, Tukey test, P>0.05)

**Table 4:** Proliferation assay shows mean population doubling number (PDN) of mesenchymal stem cells (MSCs) after one hour of treatment with 100, 1000, and 2000 µM of sodium nitroprusside (SNP) followed by 5, 10 and 15 days of incubation


Day	5	10	15
Dose (µM)			

0	1.83^a^ ± 0.02	3.57^a^ ± 0.03	4.69^a^ ± 0.01
100	1.79^a^ ± 0.02	3.53^a^ ± 0.02	4.69^a^ ± 0.04
1000	0.125^b^ ± 0.00	1.13^b^ ± 0.01	1.15^b^ ± 0.01
2000	0.25^c^ ± 0.00	0.33^c^ ± 0.02	0.65^c^ ± 0.05


Values are means ± SD. Means with the same letter code do not differ significantly from each other in a column (ANOVA, Tukey test, P>0.05).

### Morphology

**Fig.2:**
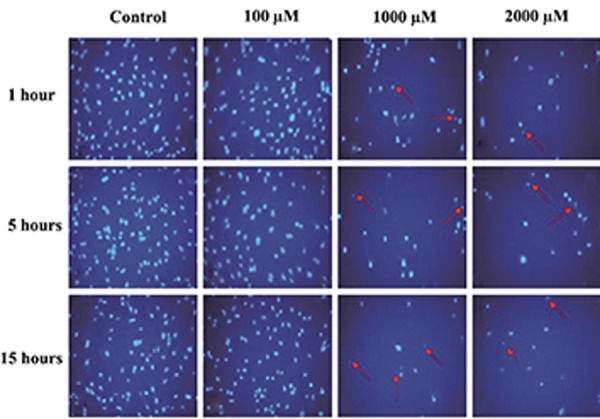
Fluorescent micrograph of bone marrow mesenchymal stem cells (BM-MSCs) stained with Hoechst, after 1, 5, and 15 hours treat- ment with 100, 1000 and 2000 µM of sodium nitroprusside (SNP). Nuclear condensation and DNA fragmentation (arrows) of cells was observed after treatment with 1000 and 2000 µM of SNP. A. Control group of the cells. Cells treated with: B. 100 µM, C. 1000 µM, and D. 2000 μM of SNP (magnification: ×200).

**Fig.3:**
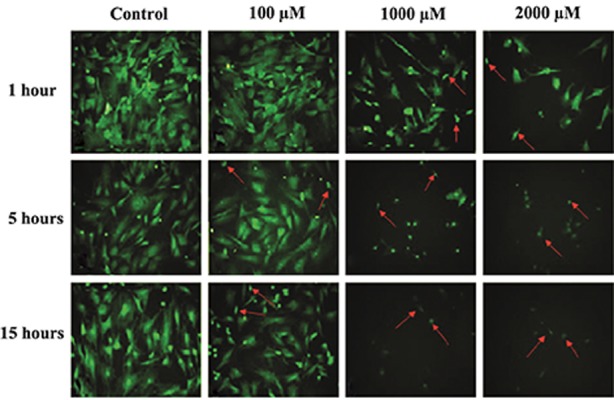
Fluorescent micrograph images of bone marrow mesenchymal stem cells (BM-MSCs) stained with acridine orange after one, 5, and 15 hours treatment with100, 1000 and 2000 µM of sodium nitroprusside (SNP). Shrinkage and complete disappearance of the cytoplasm in some cells (arrows) was observed after treatment with 1000 and 2000 µM of SNP. A. Control group. Cells treated with: B. 100 µM, C. 1000 µM, and D. 2000 μM of SNP (magnification: ×200).

Morphological study of the nuclei from MSCs treated with 1000 and 2000 µM of SNP at one, 5, and 15 hours showed chromatin condensation and nuclear breakage (Fig.2) as well as significant reduction (P<0.05) in nuclei diameter (Table 5). All concentrations of SNP at 5 and 15 hours caused remarkable changes in cytoplasmic morphology, which included shrinkage and rounding as well as complete disappearance of the cytoplasm in some cells (Fig.3). There was a significant reduction (P<0.05) in cytoplasmic area at 5 and 15 hours in the groups of cells treated with 100, 1000, and 2000 µM of SNP. Only the 100 µM concentration of SNP did not change the cytoplasm area at one hour (Table 5). There were no changes in nuclei diameters observed in cells treated with 100 µM of SNP at one, 5, and 15 hours, whereas the 1000 and 2000 µM concentrations significantly reduced (P<0.05) the nuclei diameters during all treatment periods.

**Table5:** Morphological assay shows mean diameter (μm) and cytoplasmic area of mesenchymal stem cells (MSCs) after one, 5, and 15 hours of treatment with 100, 1000 and 2000 µM of sodium nitroprusside (SNP) followed by 24 hours of incubation


HourDose (µm)	1Nucleus	Cytoplasmic	5Nucleus	Cytoplasmic	15Nucleus	Cytoplasmic
	diameter (μm)	area (µm^2^)	diameter (μm)	area (µm^2^)	diameter (μm)	area (µm^2^)

0	11.99^a^ ± 3.30	3935.29^a^ ± 17.27	11.93^a^ ± 2.7	4013.88^a^ ± 18.48	11.68^a^ ± 2.62	4066.72^a^ ± 21.82
100	11.29^a^ ± 2.7	3948.31^a^ ± 17.90	11.09^a^ ± 3.08	3504.60^b^ ± 11.82	11.06^a^ ± 3.04	2933.76^b^ ± 13.64
1000	10.26^b^ ± 3.2	3588.89^b^ ± 17.03	10.00^b^ ± 1.72	1517.98^c^ ± 8.79	8.99^b^ ± 1.84	1233.71^c^ ± 9.14
2000	10.09^b^ ± 2.4	3286.57^c^ ± 14.32	9.78^b^ ± 1.63	1174.44^d^ ± 5.39	8.32^c^ ± 1.60	799.16^d^ ± 3.49


Values are means ± SD. Means with the same letter code do not differ significantly from each other in a column (ANOVA, Tukey test, P>0.05).

### Intracellular Na^+^, K^+^and Ca^2+^concentrations

Treatment with 100, 1000, and 2000 µM of SNP caused a significant reduction (P<0.05) of intracellular Na^+^content compared to the control. We observed no alteration in intracellular K^+^concentration and a steady state was observed compared with the control group. The 1000 and 2000 µM concentrations of SNP significantly reduced (P<0.05) of intracellular concentrations of Ca^+^in a dose-dependent manner. There was no change observed with respect to the 100 µM dose (Table 6).

### Metabolic activity of the cells

Data analysis showed that activities of ALT, AST, and ALP significantly reduced (P<0.05) after treatment with 100, 1000, and 2000 µM of SNP compared with the control group. This reduction was dose-dependent. The treatment caused a significant increase (P<0.05) in LDH activity compared to the control, which was dosedependent (Table 7). 

**Table 6:** Electrolyte determination assay shows the effect of sodium nitroprusside (SNP) on intracellular Ca^2+^(mg/dl), Na^+^(µg/ml), and K^+^(µg/ml) levels after treatment with 0, 100, 1000 and 2000 µM of SNP for one hour, followed by 24 hours of incubation


Dose (µM)	Ca^2+^	Na^+^	K^+^

0	0.82^a^ ± 0.00	94.16^a^ ± 02.00	7.26^a^ ± 0.00
**100**	0.84^a^ ± 0.00	84.16^b^ ± 02.00	7.10^a^ ± 0.57
**1000**	1.01^b^ ± 0.57	72.16^c^ ± 1.15	7.76^a^ ± 0.00
**2000**	1.22^c^ ± 0.57	71.16^c^ ± 0.00	7.29^a^ ± 1.60


Values are means ± SD. Means with the same letter code do not differ significantly from each other in a column (ANOVA, Tukey test, P>0.05).

**Table 7:** Enzymatic assay shows the mean activity of AST, ALT, LDH and ALP after treatment with 100, 1000, and 2000 µM of sodium nitroprusside (SNP) for one hour followed by 24 hours of incubation


Dose (µM)	AST	ALT	LDH	ALP

0	417.61^a^ ± 1.1	317.97^a^ ± 1.03	2780^a^ ± 13.5	1273.7^a^ ± 12.4
100	305.33^b^ ± 2.5	291.79^b^ ± 0.66	3417^b^ ± 8.3	1157.8^b^ ± 16.62
1000	67.27^c^ ± 0.4	258.40^c^ ± 3.40	3831^c^ ± 35.6	1041.3^c^ ± 16.5
2000	48.31^d^ ± 1.3	277.52^d^ ± 0.47	4179^d^ ± 74.2	907.56^d^ ± 12.4


Values are means ± SD. Means with the same letter code do not differ significantly from each other in a column (ANOVA, Tukey test, P>0.05). AST; Aspartate transaminase, ALT; Alanine transaminase, LDH; Lactate dehydrogenase, and ALP; Alkaline phosphatase.

## Discussion

Osteoporosis is a worldwide problem, particularly in industrial societies. Since NO is synthesized in the osteoblasts (23), thus an understanding of the effects of low dose SNP as a NO releasing agent on MSCs, which are considered to be the osteoblast cellular backup, may open a new avenue for osteoporosis prevention or treatment. In their previous study, Chu et al. (21), have shown that treatment of adult mouse bone marrow multipotent progenitor cells with high doses (500 to 2000 µM) of SNP significantly reduced cell numbers after 48 hours, where as they observed no significant change with respect to the 100 μM treatment after 48 hours. In another study, Felka et al. (4) investigated the effect of SNP (100 to 2000 µM) on human BM-MSCs. They found that SNP caused a concentration dependent reduction in respiratory activity over 24 hours. In the above studies, although the cell lines were similar, the results were not identical. In addition to NO, it has been determined that SNP would also release CN. Release of NO from SNP takes place in less than one hour (22), therefore SNP treatment for a longer period of time would release more CN (10). The previous studies were carried out with 24 and 48 hours of SNP treatment, therefore it might be assumed that the toxicity was due to inhibition of the mitochondrial respiratory chain due to the lengthy exposure. In the present study, the main goal was to determine short time treatment of MSCs with SNP in order to investigate any possible effect and its mechanism.

Trypan blue staining results showed no change in viability of MSCs treated with 100 µM of SNP at 1 and 5 hours compared to the control, whereas after 15 hours viability has significantly reduced. Trypan blue is a dye that cannot pass through an intact cell membrane and enter the cell when the membrane is damaged (24). Chu et al. (21) also used trypan blue staining to investigate viability, where they found no cell membrane damage after 48 hours of treatment with 100 µM of SNP which has differed from our result. Crystallization of yellow formazan is due to its reduction by dehydrogenase enzymes such as succinate dehydrogenase (complex II) which is a component of the electron transfer chain (ETC) (25). As previously noted, SNP can liberate CN and this molecule is an inhibitor of complex IV of the ETC which subsequently causes inhibition of ATP production (10). Therefore, inhibition of mitochondrial respiration might be a reason for reduction in viability based on the MTT assay. Our results according to the MTT assay might show that at 5 and 15 hours the 100 µM concentration of SNP interfered with cellular respiration and ATP production which agreed with the findings by Felka et al. (4), but not over a short time (one hour) where we observed no change in viability. Previous study in addition to the current study have shown that concentrations higher than 100 µM are toxic at all treatment periods, which mainly may be due to CN production.

Investigations in our study proved that treatment with low concentrations of SNP over a short time (one hour) had no adverse effect on viability, therefore we assumed that a low concentration and short time period might be advantageous to cells. We performed additional experiments to investigate this hypothesis.

It is well known that NO is a signaling molecule in the cell which affects numerous cellular characteristic such as cell proliferation and metabolism (26, 27). In the present study, we have observed that cell viability was not the only factor affected by high concentrations (1000 and 2000 µM) of SNP. The significant reduction of MSC proliferation potential based on significant decrease of PDN and colony forming ability were estimated. Our results supported those by Chu et al. (21) and Tanner et al. (28) where they studied adult mouse bone marrow multipotent progenitor cells and human vascular smooth muscle cells. Treatment with 100 µM of SNP for one hour showed no effect on PDN even after 5, 10, and 15 days of incubation. This finding indicated that low concentration of SNP did not have any impact on cellular mechanisms or proliferation. Estimation of colony forming ability also supported the PDN, where we have observed no change in the diameters of the colonies after one hour of SNP treatment followed by 7 and 14 days of incubation. Colony diameter is a factor which shows the ability of a cell to proliferate and produce daughter cells. The changes in the number of colonies after treatment with 100 µM for one hour and incubation for 7 and 14 days might be due to impairment of cellular metabolism. In the present study treatment of the cells with SNP (100, 1000, and 2000 µM) caused significant concentration dependent reductions in ALT, AST, and ALP activities, whereas LDH activity increased in a concentration dependent manner. Here we might refer to the Warburg effects to explain the metabolic changes (29). The cell should generate energy by oxidative breakdown of glucose in the presence of sufficient oxygen. However, due to metabolic changes from aerobic to anaerobic metabolism, lactic acid would be generated with subsequently less ATP (13). This situation would bring about poor energy production and insufficient ATP to support cellular activity. The same effect might have happened with MSCs in this investigation, where during the course of time, low concentrations and short treatment times might cause cellular mechanism impairment to the extent which it affected the number of cells for colony formation, diameters which is a matter of cell proliferation and viability. Chen et al. (15) mentioned that treatment of osteoblasts with SNP significantly increased LDH activity which was a good comparison with the results of the current study of a metabolic shift from aerobic to anaerobic.

Another important characteristic of the cell is its morphology which may change due to reasons such as nuclear, chromatin, and cytoskeleton damage. Overproduction of NO is considered to result in free radicals (15, 30, 31) and it has been shown to induce apoptosis at concentrations higher than physiologic levels (32, 33). Thus, in this study, we have investigated cell morphology under the influence of SNP. Treatment of cells with 100 µM of SNP for one hour caused no change in cell morphology, whereas treatment at 5 and 15 hours showed significantly reduced cytoplasms. High concentrations (1000 and 2000) at any treatment period significantly reduced the nuclear diameter and cytoplasm area, which agreed with studies carried out by Seo et al. (34), Chen et al. (14), and Park et al. (35). Reduction of cytoplasm area due to treatment with 100 µM of SNP could be based on inhibition of protein assembly which might be due to Ca^2+^imbalance and low energy production in the cell.

Elevation of Ca^2+^was related to induction of apoptosis (36), since we found no alteration in Ca^2+^concentration following one hour treatment with 100 µM of SNP. Therefore, no change in viability or PDN could be expected. However we could not explain the reduction in cytoplasmic area and colony number with respect to Ca^2+^content and longer treatment times (5 and 15 hours). Elevation in Na^+^irrespective of the steady level of K^+^ might be considered as a reason to partially explain the membrane potential imbalance which could explain the membrane abnormality (37) and cytoskeleton miss-arrangement (38). We observed the toxic effects of high concentrations (1000 and 2000 µM) of SNP on electrolyte levels. The results showed elevated intracellular Ca^2+^and significantly reduced Na^+^concentration. Therefore, in addition to a metabolic shift from aerobic to anaerobic, an electrolyte imbalance also could have a cumulative effect on viability and proliferation ability. 

## Conclusion

High doses of SNP as an NO releasing agent over a long period of time are not advisable. However short term use of low doses (less than 100 µM) may be safe. We must consider that low doses of SNP can also have negative effects on biochemical mechanisms. Therefore, use of alternative NO releasing agents may be recommended for therapeutic purposes and other investigations. However, in order to confirm this conclusion, we recommend additional *in vitro* and *in vivo* investigations. 
